# Small bowel exteriorisation after uterine perforation from manual vacuum aspiration for abortion in a young cameroonian: a case report

**DOI:** 10.11604/pamj.2016.25.198.10006

**Published:** 2016-11-28

**Authors:** Efuetnkeng Bechem, Djokam Leopold, Takang William Ako

**Affiliations:** 1 Bamenda Regional Hospital, Bamenda, Cameroon; 2 Faculty of Health Sciences, University of Bamenda, Bamenda, Cameroon

**Keywords:** Manual vacuum aspiration, uterine perforation, small bowel, abortion

## Abstract

Manual vacuum aspiration is an effective and safer surgical method of uterine evacuation for an abortion. Nonetheless, it can present some life-threatening complications like uterine perforations. In a uterine perforation the suction cannula is thought to be usually involved in the perforation and the resulting intraabdominal organ damage. We presented a case of a young muilti-parous Cameroonian woman who was underwent a manual vacuum aspiration for a first trimester incomplete abortion, and which was complicated by a fundal uterine perforation with exteriorisation of small bowels through the vagina.

## Introduction

Abortion and its complications are serious problems in gynaecology. Both spontaneous and induced abortion may have life threatening complications. Surgical management of abortions are known to have some serious complications especially with the use of uterine curettage [1]. With the advent of manual vacuum aspiration (MVA), lesser complications were reported and this method has proven its effectiveness and safety [2]. Despite this safety of the MVA, cases of life-threatening complications have been reported. We are presenting a case of small bowel exteriorisation after uterine perforation from manual vacuum aspiration for incomplete spontaneous abortion.

## Patient and observation

**Figure 1:**
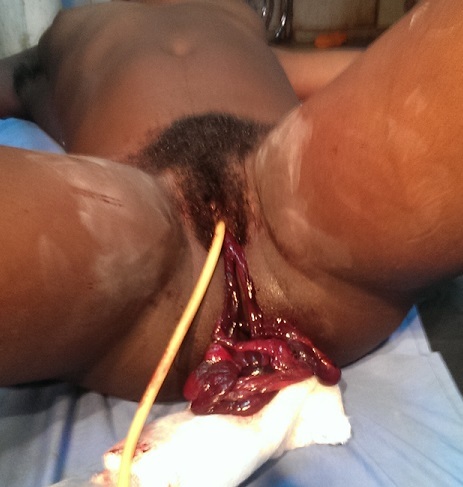
Small bowel exteriorised through the vagina

**Figure 2:**
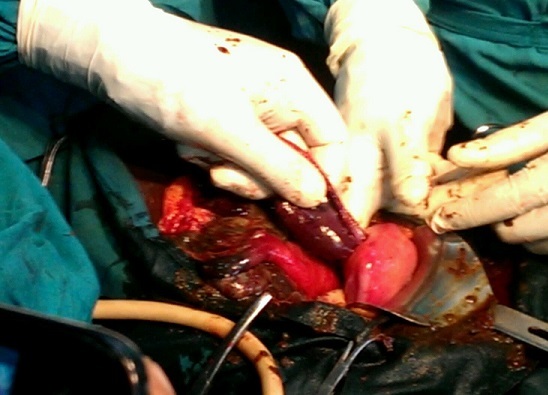
Laparotomy section showing the small bowel getting into the perforated uterus

A 24 year old G2 P1 011 was referred to the Bamenda Regional Hospital from a district hospital for the management of exteriorisation of bowel through the vagina. She was seen earlier that day for per vagina bleeding accompanied with mild lower abdominal pain, without any relevant past history. After clinical assessment she was diagnosed of incomplete abortion in an eleven week pregnancy, and indicated for uterine evacuation by manual vacuum aspiration. Upon aspiration, the student doctor noticed that bowel was coming from the uterus. He immediately stopped and referred her to the Regional hospital for management. Upon arrival at the Regional Hospital, she had a good general condition and normal vital parameters, her abdomen was also soft. On inspection of the vulva, small bowel was exteriorised which was worsen by increased intraabdominal pressure (Figure 1). Our working diagnosis was uterine perforation with small bowel exteriorisation. The patient was cross-matched 2 pints of whole blood and taken immediately to the theatre for an emergency laparotomy. We found a 2cm uterine perforation at the fundus with small bowel prolapse (Figure 2). She benefitted from a resection of 190 cm of small bowel with termino-terminal anastomosis. Uterine debris was aspirated per abdominally followed by closure of the uterine rent. The abdomen was washed with warm saline and intraabdominal drained placed at the Douglas pouch and left para-colic gutter. The post operatory was complicated by wound infection which was managed accordingly and by patient discharged on post operatory day 17.

## Discussion

Abortion is the termination of pregnancy before viability, and in developing countries viability is attained after 28 completed weeks. There are spontaneous and induced abortions, and spontaneous abortions can be a threat, inevitable, incomplete, complete, missed or habitual abortions. Abortion is a serious condition in obstetrics and gynaecology. In Cameroon global figures on abortion are absent but a recent study reported that 26.3% of women attending obstetrics/gynaecological clinics have had a voluntary induced abortion [3]. Abortion is one of the main causes of maternal death worldwide and especially in sub Saharan Africa. In a systemic analysis, Say et al found abortion to be the forth direct cause of maternal death (8%) after haemorrhage (27%), hypertensive disorders (14%), and sepsis (11%) [4]. According to this review abortion was still forth in Sub-Saharan Africa but represented 10% of all maternal death. In Cameroon, a recent study at the Yaoundé Teaching Hospital reported unsafe abortion to be the second cause of maternal death (25%), after haemorrhage [5]. The management of incomplete abortion has evolved from dilatation and curettage to manual vacuum aspiration, although in some cases curettage is still used. This evolution was due to the numerous complications following uterine curettage. Immediate complications of uterine curettage include: haemorrhage, uterine perforation, and Ashermann syndrome [6]. Manual vacuum aspiration has credit of lesser complications and ease of use [7]. Westfall et al in 1998 assessed the safety and effectiveness of manual vacuum aspiration (MVA) for abortion in in a primary care setting [8]. They found a case of uterine perforation out of 1,677 MVAs done (0.06%). Mittal in a similar study in 1985 evaluated 9344 case of MVA for first trimester abortions and had 37 cases of uterine perforations (0.39%) [9]. He found out that 50 % of the perforations were due to the suction cannula and that fundal and anterior walls perforations were common. In his paper Mittal reported that all cases of perforations were in multiparous women, like in our case. Recent articles on MVA causing uterine perforations are rare in literature. Uterine perforation is associated with life threatening complications like intraabdominal haemorrhage and injury to viscera (bladder, intestines) [10]. We had a multiparous girl who had a spontaneous incomplete abortion. Upon suction her uterus was perforated with bowel exteriorisation. This explains the findings by other authors that uterine perforation is possible with MVA. Her bowel was exteriorised and that can be explained by the suction grip of the cannula and pulled the bowel through the uterine rent to the vagina.

## Conclusion

Manual vacuum aspiration has been reported be an effective and safely method of uterine evacuation following an abortion. Some studies have reported uterine rupture following the use of MVA usually due to the suction cannula. Severe injuries such as bladder and bowel injury can occur following these perforations. We just reported a case of small bowel exteriorisation from uterine perforation using manual vacuum aspiration for an incomplete abortion.
